# Effect of Different Wax Pattern Manufacturing Techniques on the Marginal Fit of Lithium Disilicate Crowns

**DOI:** 10.3390/ma15144774

**Published:** 2022-07-07

**Authors:** Huda Ahmed Alshehri, Sara Mohammed Altaweel, Raghdah Alshaibani, Esraa Ahmed Alahmari, Hanan Nejer Alotaibi, Afnan Fouzan Alfouzan, Nawaf Labban

**Affiliations:** Department of Restorative Dental Sciences, College of Dentistry, King Saud University, P.O. Box 60169, Riyadh 11545, Saudi Arabia; staweel-ksu@hotmail.com (S.M.A.); raghdahs@bu.edu (R.A.); esraa.alahmari@gmail.com (E.A.A.); haalotaibi@ksu.edu.sa (H.N.A.); afnan477@hotmail.com (A.F.A.); nalabban@ksu.edu.sa (N.L.)

**Keywords:** marginal fit, wax pattern, Cad/CAM, ceramic crown, 3D-printing, lithium disilicate

## Abstract

Purpose: The present study evaluated the marginal gap of lithium disilicate crowns fabricated through three different wax pattern techniques; Conventional, Milling and 3D-printing. Materials and Methods: Thirty stone models were replicated from a stainless-steel model representing a prepared tooth; ten were sent to make conventional wax patterns while the remaining were sent to a digital dental scanner. The computer aided design was completed and STL (Standard Tessellation Language) files were sent to either milling or 3D-printing machines. All wax patterns (*n* = 30) were pressed, and a stabilizing instrument was used to secure the crowns on the master model. The marginal gap was measured at 18 points for each crown using a digital microscope (µm) (*n* = 540) and compared using One-way ANOVA (*p* ≤ 0.05). Results: There was a significant difference in the marginal gap value between all three groups (*p* < 0.01) where the milled group showed the least mean gap (28.87 ± 30.18 µm), followed by 3D printed (47.85 ± 27.44 µm), while the highest mean marginal gap was found in the conventional group (63.49 ± 28.05 µm). Conclusion: Milled and 3D-printed wax patterns produced better fitting crowns compared to conventional techniques.

## 1. Introduction

The major determining factors for a successful clinical performance of fixed dental prosthesis are high marginal accuracy and an adequate internal fit [1]. Increased marginal discrepancies lead to a higher rate of cement dissolving due to its exposure to oral fluids and chemomechanical dissolution in the oral cavity [2]. Consequently, the longevity of the restored tooth will be compromised by the increased risk of plaque retention, caries and pulpal pathology [3]. A marginal fit between 25 and 40 µm for cemented restorations has been suggested as a clinical goal, but these levels are rarely achieved [1]. Some studies indicated that a marginal fit of ≤120 µm is clinically acceptable [4], but a more recent study has concluded that a marginal fit of ≤100 µm is more suitable [5].

Methods used for measuring marginal adaptation include direct inspection by optical microscopy, view of the cross-section of cemented crowns, and indirect inspection via impression replica technique, scanning electron microscopy and dye penetration methods [6]. Direct inspection by optical microscope is ought to be repeatable, less invasive and does not include intermediate material between crown and its substrate.

The gold standard over the last few decades for posterior teeth rehabilitation was cast gold restorations, due to its favorable long-term success. Advantages of all-ceramic crowns over metal ceramic include esthetics, improved functionality and long-term survival compared to or superior to metal-ceramic crowns [7]. Recently, various all-ceramic systems and manufacturing processes have been introduced to the dental market and have become popular due to their improved physical properties and the increased demand for esthetic dental restorations [8]. These include powder-liquid, pressed and machined ceramics processing technique [9]. The pressed lithium disilicate crowns are considered the material of choice for restorations due to their excellent esthetics, adequate marginal adaptation and the possibility of adhesive cementation [10].

A critical step in making all ceramic crowns through hot pressing is the fabrication of the wax pattern. Wax has numerous limitations including thermal sensitivity, elastic memory, and high coefficient of thermal expansion [11]. A handmade natural wax pattern is usually used to fabricate lithium disilicate restoration; this conventional method requires meticulous technique that involves many human-related factors that might cause errors in crown fabrication [12,13,14].

Currently, the fabrication of the wax pattern has been made with different computer-aided design/computer aided manufacturing (CAD/CAM); either subtractive by milling or additive by three-dimensional (3D) printing, reducing many limitations of the conventional waxing technique [15].

Several studies were conducted to compare the fit of lithium disilicate crowns fabricated directly by milling ceramic blocks to those fabricated by conventional wax pattern technique [16,17,18]; but there is lack of information in the current literature on the adaptation quality of lithium disilicate crowns produced from milled and 3D printed wax patterns to conventional ones. 

For the above-mentioned perspectives, we conducted this study to compare the marginal fit of lithium disilicate crowns pressed through wax patterns fabricated by milling, 3D printing versus the conventional waxing techniques.

### Rational

Lithium disilicate crowns are mostly used in the fabrication of fixed prosthesis due to their high esthetic and physical properties. However, different techniques in preparing wax pattern for lithium disilicate crowns could affect their marginal accuracy. This research hypothesis is that there is a difference between gap measurements of ceramic crowns produced by three different ways of wax pattern fabrication.

## 2. Materials and Methods

The study was designed to evaluate the marginal adaptation of lithium disilicate crowns (IPS E-max©, Ivoclar Vivadent, Schaan, Germany) made from wax patterns fabricated by three different manufacturing techniques; conventional wax pattern, milling, and 3D-printing. 

**Figure 1:**
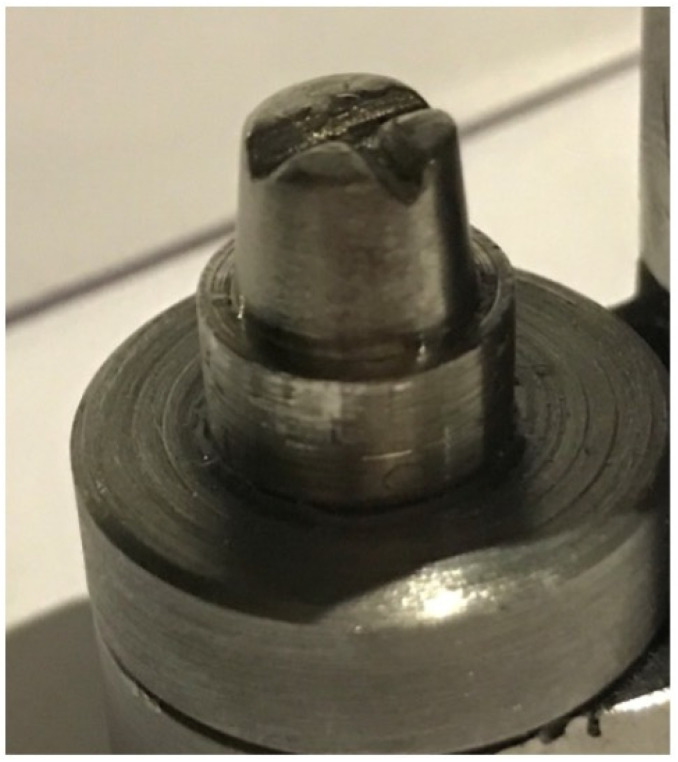
Master model.

A cylindrical, machined stainless-steel die was fabricated to serve as a master die. The die represents a first molar complete crown preparation with 6 mm height, 10 mm diameter at the finish line and 1 mm shoulder margin preparation with axial wall taper of 5 degrees. A v-shaped groove was placed on the occlusal surface of the die to prevent rotation during seating (Figure 1).

**Figure 2:**
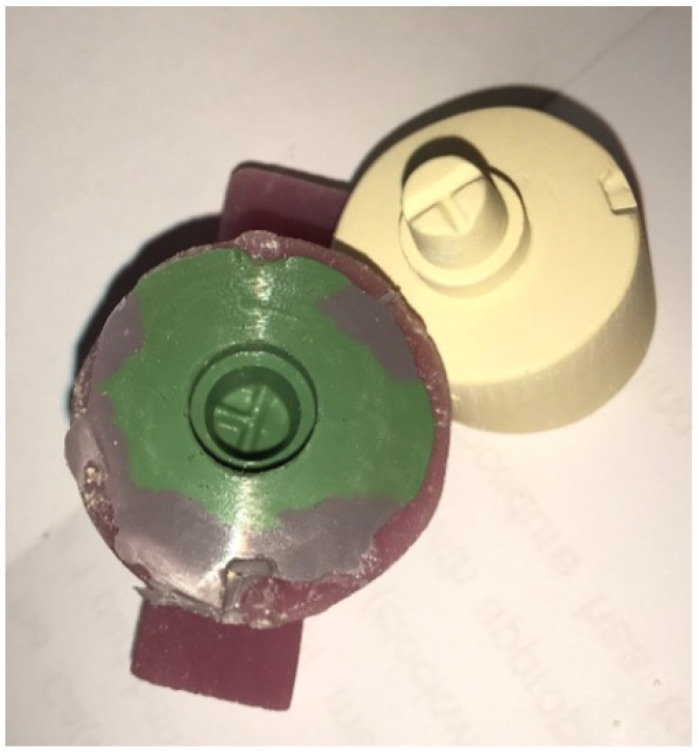
Impression and stone model.

Thirty custom trays were fabricated with light-cured acrylic resin (Preci Tray, Yeti Dental, Engen, Germany). Polyvinyl siloxane impression material was used (Express, 3M ESPE, United states) to make thirty impressions of the standard die. Impressions were poured with type IV gypsum (YETI Rock IV, Yeti Dental, Engen, Germany) (Figure 2). After complete setting, models were divided randomly into 3 groups of 10 specimens in each group.

**Figure 3:**
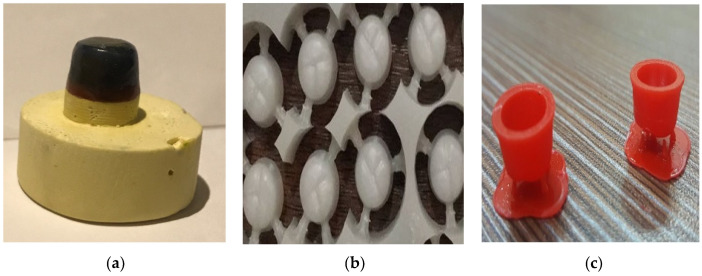
Three different types of wax patterns; conventional (**a**), Milled (**b**) and 3D-printed (**c**).

- Wax pattern fabrication:

Group 1—Conventional wax pattern Technique:

After application of two layers of die-spacer (Aqua-Fit, Renfert, Hilzingen, Germany), wax pattern in the lab was made for 10 stone models using conventional methods by inlay wax (Crowax, Renfert, Hilzingen, Germany) (Figure 3a).

**Figure 4:**
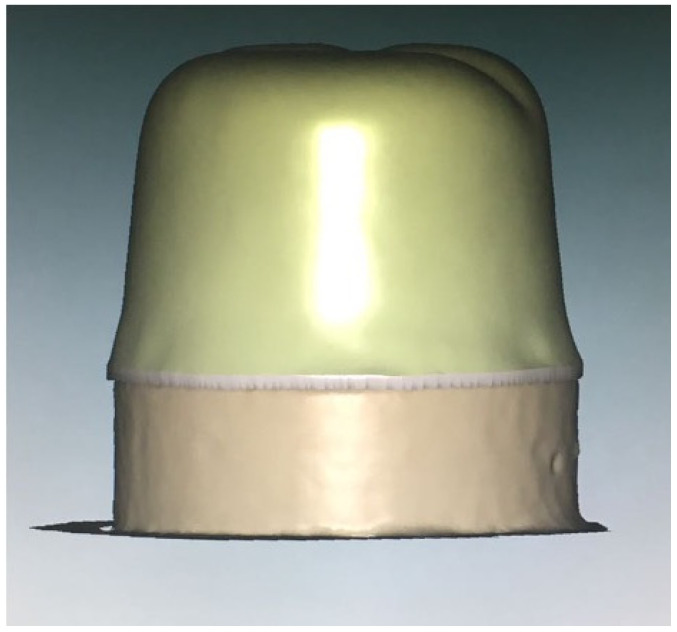
CAD model.

Group 2-CAD/CAM by milling:

Ten stone models were scanned using the digital scanner (Ceramill Map 400, Amman Girrbach, Vorarlberg, Austria) and designed using CAD software (Ceramill Mind, Amman Girrbach, Vorarlberg, Austria) (Figure 4). After that, the design was used with the 5-axis milling machine (Ceramill motion 2, Amman Girrbach, Vorarlberg, Austria) using castable wax material (Ceramill^®^ D-wax, Amman Girrbach, Vorarlberg, Austria) (Figure 3b).

Group 3—3D printing:

The same way of scanning and designing used in group 2 was used here, but the design was sent in a digital format (STL) through email for printing a wax pattern using the material (Visijet crystal) with a 3D printing machine (Projet 3500HDmax, 3D Systems, Inc., Rock Hill, SC, USA) (Figure 3c).

B. Crowns Fabrication:

All 30 wax patterns from the three groups were sent to the lab to be invested using standard heat pressing technique using a cylindrical rubber ring with a phosphate-bounded material (IPS PressVEST Speed, Ivoclar Vivadent, Ellwangen, Germany). Then, the wax was burned out in a burning machine (Miditherm 100 MP, BEGO, Bremen, Germany). Finally, the ceramic crowns were fabricated through heat pressing machine (Programat EP 5000, Ivoclar Vivadent, Germany) using ceramic ingots (E.max Press, Ivoclar Vivadent, Germany). All procedures were conducted with one dental technician.

**Figure 5:**
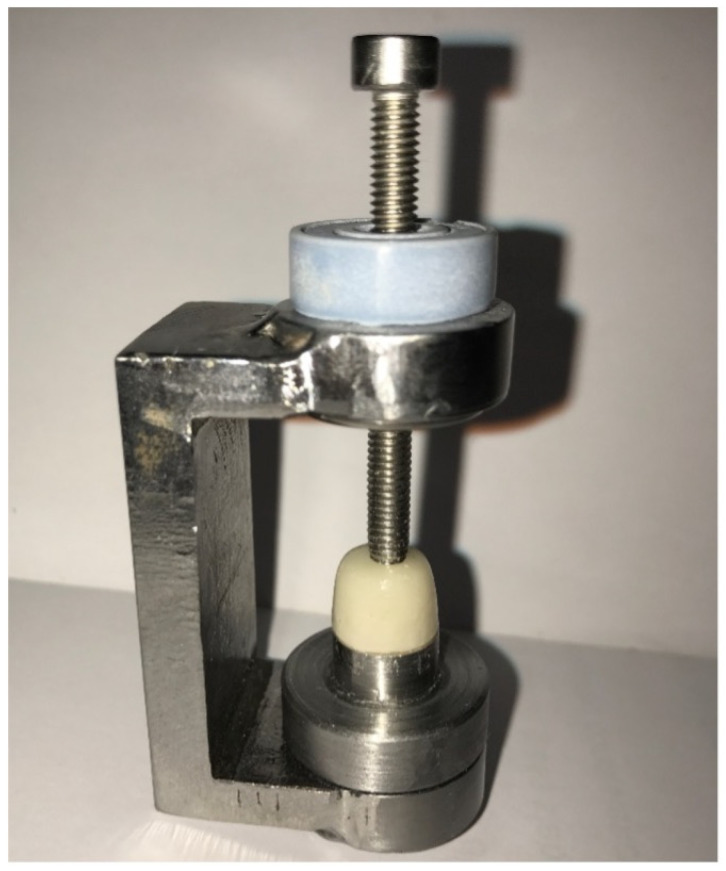
The stabilizing instrument.

C. Fitting procedure:

A stabilizing instrument was used to secure the crown on the master model (Figure 5). Eighteen points were located around the margins of the master model as a standard reference that will guarantee to measure all crowns at the same points. The Digital microscope was used for measurements of the marginal gap in micrometers (µm) × 150 (KH-7700 Hirox Co., Ltd., Tokyo, Japan). Calibration was made between two operators for the gap measurements and the interexaminer reliability was 99%.

Statistical Analysis:

Descriptive statistics are stated for all gap measurements. One-way ANOVA analysis of variance followed by multiple range comparison test post hoc (Dunnett T3) (α = 0.05) using statistical software (SPSS 16.0 for windows; SPSS, Inc., Chicago, IL, USA).

## 3. Results

### 3.1. Descriptive Statistics

The mean marginal gap (µm) and the standard deviation of all groups are presented in Table 1. Mean marginal gap was highest in the conventional group (63.49 ± 28.05 µm) followed by (47.85 ± 27.44 µm) and the least marginal gap was in the milled group (29.87 ± 30.18 µm).

High variance was noticed among the samples, but since the sample size in each group was 180 readings, then by using central limit theorem, the normality for $\bar{x}$ was considered fulfilled. Therefore, the square root of the mean and standard deviation was used to reduce variance (X^2^). Values were used for multiple comparison test.

### 3.2. Comparison of Marginal Gap among the Groups

One-way Analysis of variance (ANOVA) was used to compare the marginal gap between the conventional, milled and 3D printed samples, there was statistically significant difference between all three groups (*p* < 0.001) as shown in Table 2.

Post hoc (Dunnett T3) was used to show the mean difference and the significance between the three groups as illustrated in Table 3.

The result of this study showed that the best marginal fit was achieved with the milled group, with the mean marginal gap of 28.87 µm ± 30.18, followed by the 3D printed group with a mean gap of 47.85 µm ± 27.44, while the conventional group showed the highest mean marginal gap of 63.49 µm ± 28.05.

## 4. Discussion

The marginal fit is considered one of the most important criteria for the long-term success of all-ceramic restorations. Increased marginal discrepancies lead to microleakage, a higher rate of cement dissolving, therefore increasing the susceptibility for caries around the tooth leading to restoration failure [2]. The significance of the marginal fit arises from the belief that secondary caries and loss of retention are the leading reasons of restoration failure [19], both of which are directly related to luting cement breakdown and marginal adaptation deficits. 

The aim of this study was to compare the marginal fit of lithium disilicate crowns pressed through wax patterns fabricated by milling; 3D printing versus the conventional techniques. The data obtained in this study show statistically significant differences between gap measurements of ceramic crowns produced by three different wax pattern manufacturing techniques, thus supporting the study hypothesis and rejecting the null hypothesis. 

Many variables were present in this study, which could affect the accuracy of fit of the wax patterns, including the technician’s skill. For that reason, only one dental technician made all the wax patterns in the conventional group, as well as all the ceramic restorations. Gap measurements were taken by two operators, where calibration was carried out prior to the experiment and the interexaminer reliability was found to be high. 

There is disagreement in the literature on the acceptable marginal gap range. The clinical goal for the marginal fit of cemented restorations is between 25 and 40 µm, however, these levels are difficult to obtain [1]. Several studies have indicated that the clinically acceptable marginal gap is ≤120 µm [4], moreover, the recent studies suggested that a marginal gap of ≤100 µm is more applicable clinically [5]. The results of the current study show that there is a significant difference in the marginal gap between all three groups (*p* < 0.001). The milled groups showed the lowest mean marginal gap, which was 28.87 µm, followed by the 3D printed group with 47.85 µm, while the conventional group showed the highest mean marginal gap of 63.49 µm. Despite the differences, all three groups fell below the clinically acceptable range with the highest mean gap of 63.49 µm in the conventional group.

The conventional method has been used for decades for fabrication of fixed prosthesis with proven long-term survival. To create an accurate fitting prosthesis, careful material selection and meticulous fabrication procedures are essential to compensate for the expansions and contractions of the different materials involved. However, due to the difficulty in controlling all the variables, combined with a tendency for human error, a poor marginal fit can result [17]. It was reported in the literature that the removal of the wax pattern from the die causes an average of a 35 µm opening of the shoulder margin [11].

The use of CAD/CAM for production of dental restorations is getting more and more popular due to the convenience, digitization, time efficiency and reduction in errors. Thanks to enhanced scanning procedures, the accuracy of digital impressions and milled restorations is high. In this study, the milled group showed the highest accuracy with the least marginal gap (28.87 µm ± 30.18). In 2018, a systematic review by Papadiochou et al. showed that CAD/CAM milled crowns were equally accurate to pressed and casted metal crowns [20].

Only few studies were conducted to compare the effect of wax patterns made by CAD/CAM on the resulting crown accuracy. Shamseddine et al. compared the fit of ceramic crowns fabricated via milled versus conventional wax patterns and found that milled wax patterns gave superior fit compared to conventional technique [21]. On the other hand, another study by Shamseddine et al. compared the fit of pressed crowns fabricated from two CAD-CAM wax pattern process plans, additive and subtractive. The results showed no significant differences between the two CAD-CAM manufacturing processes (*p* > 0.05) (mean marginal gaps were 105.1 µm ± 39.6 in the milled patterns and 126.2 µm ± 25.2 for the additive process) [22]. The current study agrees with the former study where milled patterns gave crowns with better fit compared to the conventional, while it was in disagreement to the later study, in which milled patterns gave also crowns with a superior fit compared to 3D-printed ones. However, in both mentioned studies the mean marginal gaps were high and could be considered clinically unacceptable. This may be due to the use of a replica technique, in which the silicone replica is coated with copper to be measured under a scanning electron microscope, unlike the current study were direct measurement took place.

The effect of the wax pattern fabrication technique on the fit of cast metal copings was reported. Vojdani et al. studied the marginal and internal fit of metal copings cast from wax patterns fabricated by milling and conventional wax pattern manufacturing techniques and showed that the milled group had significantly larger marginal gaps (157.37 ± 20.63 µm) than the conventional group (69.54 ± 15.60 µm) [23]. This result however could not be compared to the current research due to involvement of different factors in casting metal. 

A limitation of this study was the use of three different types of wax pattern in the fabrication process: (Visijet crystal) for the 3D printing; (Ceramill^®^ D-wax, Amann Girrbach AG, Koblach, Austria) for the milled; and inlay wax for the conventional group, where all have different inherited properties. However, this could not be avoided due to the different manufacturing recommendations. In this study, we used one system for the milling and one system of the 3D printing. Future studies should compare different milling and 3D printing machines. Further studies with more sample size are needed.

## 5. Conclusions

Within limited sample size, the marginal gap of pressed lithium disilicate crowns fabricated from milled wax pattern were superior when compared with the 3D printed and conventional waxing, the latter showing the highest marginal opening. The digital methods seem to be a legitimate alternative to the traditional methods of wax fabrication.

## Figures and Tables

**Table 1:** Mean marginal gap of crowns fabricated through conventional, milled and 3D printed wax patterns (µm).

Crown Type	Minimum	Maximum	Mean	Std. Deviation
Conventional	1.27	99.13	63.49	28.05
Milled	1.27	99.13	29.87	30.18
3D printed	3.81	97.93	47.85	27.44

**Table 2:** Multiple comparison test between conventional, milled and printed groups.

	Sum of Squares	df	Mean Square	F	Sig.
Between Groups	760.152	2	380.076	68.861	0.000
Within Groups	2963.964	537	5.519		
Total	3724.116	539			

**Table 3:** Mean difference and One-way ANOVA between the three groups.

	Conventional	Milled	3D Printed
Conventional		2.88 *	1.08 *
Milled	−2.88 *		−1.79 *
3D printed	−1.08 *	1.79	

* The mean difference is significant at the *p* < 0.001 level.

## Data Availability

Data available on request from corresponding author.
